# Individual Differences in Children’s Preference to Learn From a Confident Informant

**DOI:** 10.3389/fpsyg.2019.02006

**Published:** 2019-09-03

**Authors:** Aimie-Lee Juteau, Isabelle Cossette, Marie-Pier Millette, Patricia Brosseau-Liard

**Affiliations:** Childhood Thinking Laboratory, School of Psychology, University of Ottawa, Ottawa, ON, Canada

**Keywords:** child development, cognitive development, selective learning, individual differences, confidence

## Abstract

Past research has demonstrated that children can use an informant’s confidence level to selectively choose from whom to learn. Yet, in any given study, not all children show a preference to learn from the most confident informant. Are individual differences in this preference stable over time and across learning situations? In two studies, we evaluated the stability of preschoolers’ performance on selective learning tasks using confidence as a cue. The first study (*N* = 48) presented children with the same two informants, one confident and one hesitant, and the same four test trials twice with a 1-week delay between administrations. The second study (*N* = 50) presented two parallel tasks with different pairs of informants and test trials one after the other in the same testing session. Correlations between administrations were moderate in the first study and small in the second study, suggesting that children show some stability in their preference to learn from a confident individual but that their performance is also influenced by important situational factors, measurement error or both. Implications for the study of individual differences in selective social learning are discussed.

## Introduction

From a young age, children prefer to learn from some individuals over others based on various characteristics, a phenomenon referred to as selective social learning (Koenig and Sabbagh, 2013). Several studies have shown that, when engaging in social learning, children use a variety of *epistemic* cues, i.e., cues indicating whether an informant likely has relevant knowledge (see Mills, 2013, for a review). For instance, children sometimes use an informant’s past accuracy (e.g., Koenig et al., 2004; Koenig and Harris, 2005) or perceptual access to information (e.g., Nurmsoo and Robinson, 2009; Brosseau-Liard and Birch, 2011) to drive their learning decisions.

Here, we focus on children’s use of one specific epistemic cue: confidence. Confidence (or lack thereof) can be expressed through body language, facial expressions, eye-gaze, and tone of voice. Confidence can be considered an indicator of knowledge: knowledgeable individuals are likely to appear confident when making claims, whereas individuals who are less knowledgeable are more likely to appear hesitant. However, confidence is an imperfect knowledge cue: Indeed, an informant’s level of confidence does not always reflect the informant’s true knowledge (Tenney et al., 2007, 2011; Huh et al., 2019). Yet, many studies have shown that, warranted or not, children on average prefer to learn from those who show confidence rather than hesitancy (Sabbagh and Baldwin, 2001; Jaswal and Malone, 2007; Birch et al., 2010; Brosseau-Liard and Poulin-Dubois, 2014; Matsui et al., 2016), and adults likewise tend to use a person’s confidence when evaluating their credibility (e.g., Whitley and Greenberg, 1986; Brewer and Burke, 2002).

To our knowledge, researchers looking at preschoolers’ propensity to learn from a confident individual over a hesitant one have to date only investigated group performance in this skill. We believe that investigating individual differences would shed important light on the use of this complex credibility cue, likely influenced by both children’s cognitive skills and socially or culturally mediated associations with expressions of confidence. However, before evaluating the substantive factors influencing a child’s understanding and use of confidence, we believe it is important to first test whether individual differences in selective learning from a confident individual are reliable.

Recently, a few researchers have begun looking at individual differences in performance between same-age children, to better explain the cognitive mechanisms underlying selective social learning. For instance, several researchers have attempted to correlate selective learning tasks with performance on theory of mind tasks (e.g., DiYanni et al., 2012; Brosseau-Liard et al., 2015), parental or familial characteristics (Reifen Tagar et al., 2014; Corriveau et al., 2016) and executive function (Jaswal et al., 2014). In these studies, individual selective learning performance is typically assessed with a single task comprising a few trials on a single occasion. However, little is known about the reliability and stability of selective learning performance.

When investigating individual differences on a certain construct, using a reliable task is essential. Variance on a psychological measure can be divided into so-called “true” variance in the psychological construct of interest and “noise” or error variance including situational variables (e.g., inattention on a specific trial). The higher the proportion of true variance to total variance, the higher the reliability of a measure. When a task is highly reliable, individual differences in scores can be used meaningfully to approximate the psychological construct of interest. However, if a task yields wildly variable performance from one situation to the next or from one administration to the next, individual performance on any given measurement occasion is unlikely to be indicative of any true underlying attribute and scores are unlikely to correlate with other measures.

Is children’s performance on a single selective learning task indicative of a stable level of ability and understanding? As mentioned above, the first decade of research into selective learning performance concentrated mainly on group-level performance and aggregate statistics (means, proportions). Selective learning tasks were thus not designed to serve as reliable measures of individual differences. Now that there is greater interest in individual performance, quantifying the stability of individual differences in selective learning performance becomes important. To date, few studies have assessed how reliable selective learning tasks are generally. A recent series of studies by Brosseau-Liard et al. (2018) has, however, demonstrated that individual preschoolers’ performance varies importantly not only between selective learning tasks based on different epistemic indicators (e.g., between a task based on an informant’s accuracy and another based on an informant’s confidence), but also between tasks based on the same epistemic indicators (i.e., two different tasks based on confidence) administered on different occasions. A recent series of studies by our research group (Cossette et al., under review) has further investigated the stability of individual differences in performance on selective learning tasks based on informants’ accuracy; this research has found that performance can be somewhat stable but is easily disrupted by task-specific factors (e.g., the order of presentation of informants across tasks and trials).

In the present manuscript, we present two studies investigating the stability of performance of preschool-age children on repeated or similar administrations of confidence-based selective learning tasks, to help determine whether such tasks, in their present form, can be used for further study of the mechanisms underlying individual use of confidence as a learning cue. In past research evaluating children’s use of confidence cues, there was always some variability between individual children’s performance; however, it is unclear how much of this variability is due to genuine differences in children’s understanding of confidence, or importance attached to this cue, and how much is due to variables (e.g., attention, idiosyncratic preferences) that can be considered measurement error.

## Study 1

Study 1 investigates the repeated performance of children on the exact same task over two visits. We thus test how consistent children are with themselves over time in their preference to learn from a more confident informant. Notably, because of the past research shedding doubt on the stability of children’s performance on selective learning tasks, we intentionally maximized the probability of obtaining consistent performance by administering completely identical tasks across a short time delay.

### Method

#### Participants

Forty-eight preschoolers (48–71 months, *M*_age_ = 60 months, 28 girls) were recruited from local daycares (*N* = 34) and an in-lab participant database (*N* = 14). The study was conducted in French. Demographic information was not collected of daycare participants, however the daycares served different neighborhoods in a large metropolitan area; the in-lab participants were primarily Caucasian with families of above-average income. Five additional participants were excluded from the final sample because they were not available for a second visit (*N* = 4) or experimenter error (*N* = 1).

#### Materials and Procedure

This study consisted of two visits. We aimed for a 7-day gap between visits, however in practice, the delay varied from 4 to 49 days (median = 8 days; all but 8 participants experienced a delay of 2 weeks or less). Children were tested individually and parents or daycare personnel, if present in the room, were told not to interact with them during the study. Participants’ answers were recorded live on an answer sheet by the experimenter conducting the study and later verified from a video recording of the experimental session.

**FIGURE 1:**
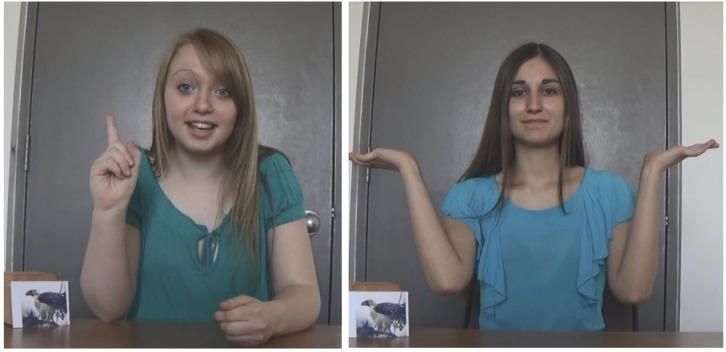
Illustration of a test trial, including confidence and hesitancy cues displayed by “I1” (**left**) and “I2” (**right**) in Form A. Written informed consent was obtained from the individuals in this figure for the publication of their images.

Both visits included the same selective learning task. This task had four versions (12 participants assigned to each) crossing two variables: task form and identity of confident informant. Both these variables were manipulated in order to ensure that findings were not dependent on a specific set of stimuli or on a specific order of presentation of confidence and hesitancy cues. Participants were introduced to two videotaped female informants, hereafter referred to as I1 and I2 (task form A) or I3 and I4 (task form B). Participants in Form A completed four trials, each centered around a picture of an animal. Children watched two videos in which each informant stated contradictory facts about the animal (e.g., one informant claiming that a bird lived in Asia and the other that it lived in Africa). One informant made her statement with confidence, expressed both verbally (“Oh, I know!” stated with assurance) and non-verbally (upright posture with shoulders back and chin high, lifting a finger in the air, facial expression of certainty). The other informant made the claim with verbal and non-verbal cues of hesitancy (“Hmm, I think …,” shoulder shrugging, puzzled facial expression, an interrogative tone and head tilting). Screenshots from one trial are presented in Figure 1. Immediately following the two videos, the experimenter asked the child what s/he thought to be the correct fact. The same procedure was repeated on all four trials. I1 spoke first on trials 1 and 2 and second on trials 3 and 4.

Children in Form B instead saw I3 and I4 make claims about pictures of four food items (e.g., a squash-like vegetable for which one informant claimed the inside was yellow and the other claimed it was orange). The procedure was otherwise identical to Form A. All children were administered the exact same task, without any changes, on both visits. Children were given a score of one each time they sided with the confident informant and 0 when they sided with the hesitant one, for a total out of four on each visit.

### Results and Discussion

Sample means of Visit 1 (V1) and Visit 2 (V2) scores were respectively *M* = 2.35 (SD = 1.12) and *M* = 2.50 (SD = 1.03). Preliminary analyses did not find effects of task form, confident informant identity within each form, experimenter, sex or age. Children performed better in lab (*M* = 3.00, SD = 1.24) than at daycares (*M* = 2.09, SD = 0.97) on V1, but this was not replicated on V2.

We estimated population values for the means and variances of Visit 1 and Visit 2 scores and the correlation between the two using Bayesian estimation with JAGS 4.3.0 and the *rjags* package (Plummer, 2016) in R 3.5.1 (R Core Team, 2018), using a uniform prior distribution for the correlation (all values between −1 and +1 equally likely), uniform priors bound by 0 and 4 for the means and diffuse priors with lower bounds of zero for the variances. Contrary to traditional frequentist analyses, this approach assigns a probability value to different population values of the parameters. We calculated Bayesian 95% highest posterior density (HPD) credible intervals for the means and their difference. Replicating prior research, both means’ credible intervals were above two or chance (V1: [2.02; 2.68]; V2: [2.19; 2.80]) and the credible interval for the difference between the two is [−0.20; 0.50], suggesting a null or small difference in performance between visits.

**FIGURE 2:**
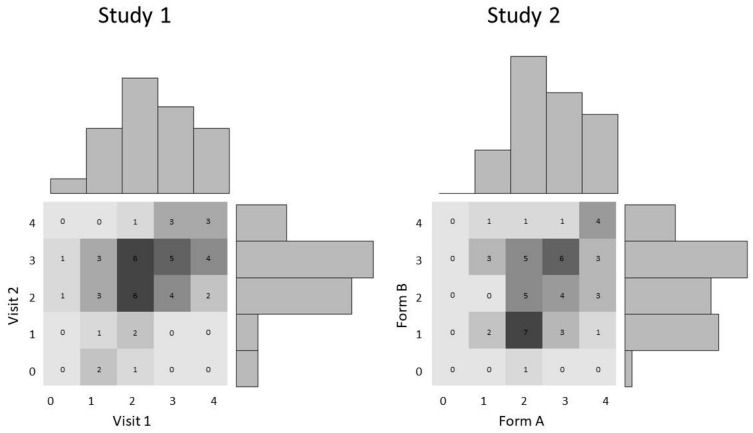
Scores (out of 4) by visit/form, Study 1 (**left**) and Study 2 (**right**). Bar graphs indicate marginal frequencies of scores on each visit/form. Numbers in the squares indicate the number of children having obtained each possible combination of scores; darker shades indicate higher frequencies.

The sample correlation between visits was *r* = 0.414; HPD interval bounds were [0.154; 0.631]. This confirms that the true correlation between the two repeated administrations is most probably positive, but there is uncertainty around its exact value, with the most probable values lying in a range typically considered “moderate.” A positive correlation indicates that there are indeed some stable individual differences in children’s performance; the size of the correlation, however, also suggests important variability between administrations. Even at the upper bound of the credible interval, a correlation of 0.631 indicates 40% of shared variance; this would be considered substantial in many contexts, but is far from spectacular for a correlation between two completely identical tasks. The fact that there did not appear to be a marked difference in performance between V1 and V2 speaks against any overall improvement from one visit to the next or, conversely, any overall reduction in performance due to boredom or other factors. Even when excluding participants with more than 2 weeks between both visits, the sample correlation was still moderate between visits (*r* = 0.491). Figure 2 presents individual patterns of responses: We do not identify any systematic pattern.

The present findings thus suggest that children *can* show stable individual differences in their preference to learn from a more confident informant. The study involved two identical tasks, thus the results could represent an upper bound on the stability of performance, as all superficial aspects of the task (e.g., how appealing each answer was, informants’ physical attributes, response tendencies such as recency bias) would influence children in the same direction on both administrations. Furthermore, it is probable that children remembered at least some of their answers from one administration to the next: Past research demonstrated that preschool-age children remember attributes of informants on similar tasks over a week-long delay (Corriveau and Harris, 2009). We conducted a second study to evaluate the stability of children’s performance on two parallel, but not identical, versions of the task. Looking at the correlation between performance on non-identical tasks eliminates the possibility of correlation inflation because children could remember their own past answers. As previous findings suggest that younger children do not perform as well on selective learning tasks (Koenig and Harris, 2005; Brosseau-Liard and Birch, 2011; Fusaro et al., 2011) and since performance did not covary systematically with age in the current sample of older preschoolers (4- and 5-year-olds) we extended our age range to include 3-year-olds in Study 2, expecting overall more individual variability across a sample with a wider age range.

## Study 2

### Method

#### Participants

Fifty preschoolers (39–70 months, *M*_age_ = 53 months, 26 girls) were recruited from local daycares (*N* = 15) and an in-lab participant database (*N* = 35) in a large metropolitan area. The study was conducted in French. As in Study 1, demographic information was not collected of daycare participants, however in-lab participants were predominantly Caucasian (nine reported a variety of other or mixed ethnic backgrounds, and five did not report ethnicity) and most families had average or above-average income. Four additional participants were eliminated because of suspected developmental delay (*N* = 2), experimenter error (*N* = 1), or sibling interference (*N* = 1).

#### Material and Procedures

The present study used the same selective learning tasks as in Study 1, with identical materials and procedures, but in a single visit. Every participant completed both forms A and B described in Study 1, one immediately after the other, counterbalancing order of forms and identity of confident informants. Between six and seven participants were randomly assigned to each of the eight possible combination of task order and each form’s informant confidence.

### Results and Discussion

Sample mean performance on form A was *M* = 2.60 (SD = 0.97) and on form B *M* = 2.32 (SD = 1.08). As the order of the forms was counterbalanced, we also calculated mean performance on the form administered first (*M* = 2.56) and second (*M* = 2.36); these were not substantially different. Preliminary analyses did not reveal effects of order of administration, identity of confident informant, or experimenter. Girls (*M* = 2.88, SD = 0.82) outperformed boys (*M* = 2.29, SD = 1.04) on form A, but this effect was not replicated on form B (and was also not evident in those administered either forms A or B in Study 1). We estimated probable population values for the form means, variances and correlation using the same specifications as in Study 1. The 95% HPD intervals for both means were above the chance value of 2 (form A: [2.33; 2.89], form B: [2.03; 2.63]), suggesting that children preferred to learn from the confident informant.

The sample correlation between children’s performance on forms A and B was positive but small at *r* = 0.242. The 95% HPD credible interval was equal to [−0.033; 0.488]. Contrary to Study 1, the credible interval in the present study includes zero, suggesting it is possible (though somewhat unlikely) that the true correlation is null. More likely is that the true correlation is small to moderate. Age was not substantially correlated with performance in either forms (A: *r* = 0.120; B: *r* = 0.065). One may wonder whether the somewhat lower correlation was due to the presence of 3-year-olds, who could conceivably be more inconsistent than older preschoolers. The sample correlation between forms for 3-year-olds (*N* = 16) was *r* = 0.093, compared to a sample correlation of *r* = 0.292 for 4- and 5-year-olds (*N* = 34), suggesting that 4- and 5-year-olds tend to have a more stable performance across task. However, this should be interpreted with caution as the sample sizes are small.

In summary, children on average preferred to learn from confident informants, consistent with the first study and previous findings. However, children’s individual scores again correlated relatively weakly on parallel tasks administered immediately one after the other. Given that the credible intervals overlap, we cannot conclude with certainty that the true correlation is lower with parallel tasks than with identical tasks. However, with a correlation that is at best moderate, the present study demonstrates that children’s performance varies between even extremely similar confidence-based selective learning tasks administered immediately one after the other.

## General Discussion

In two studies, we evaluated the stability of children’s propensity to preferentially learn from a confident informant rather than a hesitant one. In both studies, we replicated past research showing that preschoolers on average prefer to learn from confident informants. Children’s individual performance was positively correlated between two administrations of the same task (Study 1) and between two parallel versions of the task (Study 2), however the sizes of the correlations were at best moderate. Correlations of this size would likely be considered substantial if they represented the association between different but correlated constructs; however, these are correlations between literally identical tasks or between very similar tasks administered immediately one after the other, a consideration that affects the interpretation of the effect size.

On one hand, there is *some* individual consistency in children’s preference to learn from a confident individual. It would be interesting to investigate the variables affecting these individual differences. Indeed, confidence as a cue has been studied relatively little, and we still know little about how young children interpret it. Children may use cues of informant credibility in order to rationally select their preferred source of information (Sobel and Kushnir, 2013), and the weight granted to different cues may change as children develop an ability to explicitly evaluate credibility (Hermes et al., 2018b). However, attention to confidence, in both children and adults, is likely also influenced by social and cultural factors. For instance, confidence may be perceived more positively in cultures that value individual achievement and assertiveness rather than modesty and conformity, and may be evaluated differently in males and females; these are however speculative statements as these effects have not been studied in young children. The present sample is too culturally homogeneous to test for these differences: Cross-cultural research would be essential. Charting developmental trends in children’s interpretation of confidence, and investigating the situations in which confidence is granted more or less weight (e.g., Tenney et al., 2007) would lead to a better understanding of the impact of this cue on everyday learning.

However, before such theoretically motivated variations can be investigated, researchers need to develop tasks that measure individual differences in the propensity to learn from confident individuals in the most reliable way. Here, we investigated the stability of children’s responses on one specific variant of such a task, modeled after tasks used in past literature, and found relatively low stability. This is somewhat worrisome given that we had expressly designed the studies to maximize the probability of obtaining correlated results, by administering completely identical tasks twice or very similar tasks in close succession. In fact, we observed a somewhat lower correlation in Study 2, where children were administered two tasks that were similar (i.e., same wording and structure) but not identical (i.e., different informants and test items), than in Study 1, where tasks were completely identical. The difference in observed correlations could be due to sampling fluctuations or to the overall less stable performance of 3-year-olds compared to older preschoolers, but could also indicate that superficial task differences cause outsize differences in children’s responses.

At this stage, different possible explanations exist for this low stability of performance: the construct itself (i.e., children’s underlying ability and/or preference to learn from a more confident informant) could be unstable; the construct could be stable but the task unreliable; or there may genuinely be few individual differences between children on this construct. The first possibility, namely that children’s true preference to learn from a confident informant fluctuates across time and situations within a single child, has rarely been mentioned in developmental selective learning research, but should likely be given consideration. Using confidence cues depends not only on (1) noticing these cues in potential informants but also (2) associating these cues with knowledge (or status, or trustworthiness) and (3) prioritizing a learning decision based on this particular cue over any other learning strategy available to the child. The first two steps of this process are likely to depend on stable characteristics: it is unlikely that, within the few minutes that a testing session would have lasted for a participant in Study 2, their understanding of confidence cues would have developed substantially. The third step, however, may genuinely not be stable. Some documented selective learning strategies, such as appearance-based preferences (e.g., Bascandziev and Harris, 2016) or order preferences (e.g., trust the last person who spoke regardless of confidence) would have resulted in stable differences across both administrations, but other situational factors may have resulted in unstable performance. It would be important for future research to investigate how consistent preschool-age children are in their learning decisions, particularly in situations where there is more than one learning strategy available to them.

The second possibility is that there are in fact true stable individual differences in the preference to learn from a confident informant, but that the tasks use to measure this performance have low reliability. In fact, the present results are congruent with other results found by our group with an accuracy-based selective learning task (Cossette et al., under review), suggesting that the issue may be with the format of the typical selective learning paradigm, rather than something specific to the use of confidence. Of course, the present research only investigated one specific format of task; the conclusions drawn here can therefore not be generalized to all possible tasks using confidence as a cue. Hermes et al. (2018a) suggested that the typical selective learning paradigm’s structure, including the forced-choice format, could mask the cognitive processes underlying children’s selective learning. Forced-choice questions additionally allow a child to respond correctly on average 50% of the time even if they do not understand or care about the confidence cue. The reliability of tasks that do not rely on a forced-choice paradigm should be investigated; such tasks may be more reliable and perhaps better suited to the testing of individual differences. Ideally, selective learning tasks should also include more trials to increase reliability; however, in young children, concerns of fatigue, boredom, and information overload should always be considered. As an additional suggestion, instead of presenting novel information, researchers could present counterintuitive or implausible facts (Lane, 2018) to see if being presented with surprising information is more likely to reveal true individual differences in children’s learning strategies.

A third possibility is that there are genuinely few individual differences on the underlying tendency to trust a confident informant in preschool-age children, and that therefore most tasks, no matter how well-designed, would yield a high proportion of variance due to measurement error (simply because very little “true” variation exists). Such a phenomenon has been argued for in other areas of child development research, specifically in infant preferential looking paradigms (DeBolt et al., 2019). In our tasks, there did appear to be *some* individual differences that held across repeated administrations; by designing tasks better suited for the measurement of individual differences, as proposed above, future research may be able to determine how much children truly vary on this construct.

In sum, the present findings show that researchers should not simply assume that a child’s performance on one selective learning task is necessarily representative of that child’s underlying skill. It is worth pointing out that selective learning tasks were originally designed for studies investigating group-level performance. Our main conclusion from the present research would therefore be to highlight the need to develop different ways of evaluating children’s learning preferences, including methodologies that maximize task reliability for the evaluation of individual differences. Only then will it be possible for these individual differences to be properly investigated and understood.

## Data Availability

The datasets generated for this study are available on request to the corresponding author.

## Ethics Statement

This study was carried out in accordance with the recommendations of the University of Ottawa Research Ethics Board with written informed consent from parents or legal guardians of all participants. Parents or legal guardians of all subjects gave written informed consent in accordance with the Declaration of Helsinki. The protocol was approved by the University of Ottawa Research Ethics Board (The cognitive mechanisms underlying selective learning in early childhood: H04-15-11).

## Author Contributions

PB-L designed the procedures. A-LJ, IC, and M-PM contributed to the data collection. A-LJ, IC, M-PM, and PB-L contributed to the data analyses and writing of the manuscript.

## Conflict of Interest Statement

The authors declare that the research was conducted in the absence of any commercial or financial relationships that could be construed as a potential conflict of interest.

## References

[B1] Bascandziev I. Harris P. L. (2016). The beautiful and the accurate: are children’s selective trust decisions biased? *J. Exp. Child Psychol.* 152 92–105. 10.1016/j.jecp.2016.06.017 27518811

[B2] Birch S. J. Akmal N. Frampton K. L. (2010). Two-year-olds are vigilant of others non-verbal cues to credibility. *Dev. Sci.* 13 363–369. 10.1111/j.1467-7687.2009.00906.x 20136933

[B3] Brewer N. Burke A. (2002). Effects of testimonial inconsistencies and eyewitness confidence on mock-juror judgments. *Law Hum. Behav.* 26 353–364. 10.1023/A:101538052 12061623

[B4] Brosseau-Liard P. Iannuzziello A. Varin J. (2018). Savvy or haphazard? Comparing preschoolers’ performance across selective learning tasks based on different epistemic indicators. *J. Cogn. Dev.* 19 367–388. 10.1080/15248372.2018.1495219

[B5] Brosseau-Liard P. Penney D. Poulin-Dubois D. (2015). Theory of mind selectively predicts preschoolers’ knowledge-based selective word learning. *Br. J. Dev. Psychol.* 33 464–475. 10.1111/bjdp.12107 26211504 PMC4600650

[B6] Brosseau-Liard P. E. Birch S. A. J. (2011). Epistemic states and traits: preschoolers appreciate the differential informativeness of situation-specific and person-specific cues to knowledge. *Child Dev.* 82 1788–1796. 10.1111/j.1467-8624.2011.01662.x 22004452

[B7] Brosseau-Liard P. E. Poulin-Dubois D. (2014). Sensitivity to confidence cues increases during the second year of life. *Infancy* 19 461–475. 10.1111/infa.12056

[B8] Corriveau K. H. Harris P. L. (2009). Preschoolers continue to trust a more accurate informant 1 week after exposure to accuracy information. *Dev. Sci.* 12 188–193. 10.1111/j.1467-7687.2008.00763.x 19120427

[B9] Corriveau K. H. Kurkul K. E. Arunachalam S. (2016). Preschoolers’ preference for syntactic complexity varies by socioeconomic status. *Child Dev.* 87 1529–1537. 10.1111/cdev.12553 27223584 PMC5042811

[B10] DeBolt M. C. Rhemtulla M. Oakes L. M. (2019). Robust data and power in infant looking time research: Number of infants and number of trials. *Talk presented at the Society for Research in Child Development*, Baltimore, MD.

[B11] DiYanni C. Nini D. Rheel W. Livelli A. (2012). ‘I won’t trust you if I think you’re trying to deceive me’: relations between selective trust, theory of mind, and imitation in early childhood. *J. Cogn. Dev.* 13 354–371. 10.1080/15248372.2011.590462

[B12] Fusaro M. Corriveau K. H. Harris P. L. (2011). The good, the strong, and the accurate: preschoolers’ evaluations of informant attributes. *J. Exp. Child Psychol.* 110 561–574. 10.1016/j.jecp.2011.06.008 21802693

[B13] Hermes J. Behne T. Bich A. E. Thielert C. Rakoczy H. (2018a). Children’s selective trust decisions: rational competence and limiting performance factors. *Dev. Sci.* 21 1–12. 10.1111/desc.12527 28229561

[B14] Hermes J. Behne T. Rakoczy H. (2018b). The development of selective trust: prospects for a dual-process account. *Child Dev. Perspect.* 12 134–138. 10.1111/cdep.12274

[B15] Huh M. Grossmann I. Friedman O. (2019). Children show reduced trust in confident advisors who are partially informed. *Cogn. Dev.* 50 49–55. 10.1016/j.cogdev.2019.02.003

[B16] Jaswal V. K. Malone L. S. (2007). Turning believers into skeptics: 3-year-olds’ sensitivity to cues to speaker credibility. *J. Cogn. Dev.* 8 263–283. 10.1080/15248370701446392

[B17] Jaswal V. K. Perez-Edgar K. Kondrad R. L. Palmquist C. M. Cole C. A. Cole C. E. (2014). Can’t stop believing: inhibitory control and resistance to misleading testimony. *Dev. Sci.* 17 965–976. 10.1111/desc.12187 24806881

[B18] Koenig M. A. Clément F. Harris P. L. (2004). Trust in testimony: children’s use of true and false statements. *Psychol. Sci.* 15 694–698. 10.1111/j.0956-7976.2004.00742.x 15447641

[B19] Koenig M. A. Harris P. L. (2005). Preschoolers mistrust ignorant and inaccurate speakers. *Child Dev.* 76 1261–1277. 10.1111/j.1467-8624.2005.00849.x 16274439

[B20] Koenig M. A. Sabbagh M. A. (2013). Selective social learning: new perspectives on learning from others. *Dev. Psychol.* 49 399–403. 10.1037/a0031619 23437803

[B21] Lane J. D. (2018). Children’s belief in counterintuitive and counterperceptual messages. *Child Dev. Perspect.* 12 247–252. 10.1111/cdep.12294

[B22] Matsui T. Yamamoto T. Miura Y. McCagg P. (2016). Young children’s early sensitivity to linguistic indications of speaker certainty in their selective word learning. *Lingua* 175 83–96. 10.1016/j.lingua.2015.10.007

[B23] Mills C. M. (2013). Knowing when to doubt: developing a critical stance when learning from others. *Dev. Psychol.* 49 404–418. 10.1037/a0029500 22889395 PMC3810952

[B24] Nurmsoo E. Robinson E. J. (2009). Children’s trust in previously inaccurate informants who were well or poorly informed: when past errors can be excused. *Child Dev.* 80 23–27. 10.1111/j.1467-8624.2008.01243.x 19236390

[B25] Plummer M. (2016). *rjags: Bayesian Graphical Models using MCMC*. *R package version* 4–6.

[B26] R Core Team (2018). *R: A Language and Environment for Statistical Computing.* Vienna: R Foundation for Statistical Computing.

[B27] Reifen Tagar M. Federico C. M. Lyons K. E. Ludeke S. Koenig M. A. (2014). Heralding the authoritarian? Orientation toward authority in early childhood. *Psychol. Sci.* 25 883–892. 10.1177/0956797613516470 24503871

[B28] Sabbagh M. A. Baldwin D. A. (2001). Learning words from knowledgeable versus ignorant speakers: link between preschoolers’ theory of mind and semantic development. *Child Dev.* 72 1054–1070. 10.1111/1467-8624.00334 11480934

[B29] Sobel D. M. Kushnir T. (2013). Knowledge matters: how children evaluate the reliability of testimony as a process of rational inference. *Psychol. Rev.* 120 779–797. 10.1037/a0034191 24015954

[B30] Tenney E. R. MacCoun R. J. Spellman B. A. Hastie R. (2007). Calibration trumps confidence as a basis for witness credibility. *Psychol. Sci.* 18 46–50. 10.1111/j.1467-9280.2007.01847.x 17362377

[B31] Tenney E. R. Small J. E. Kondrad R. L. Jaswal V. K. Spellman B. A. (2011). Accuracy, confidence, and calibration: how young children and adults assess credibility. *Dev. Psychol.* 47 1065–1077. 10.1037/a0023273 21443337

[B32] Whitley B. E. Jr. Greenberg M. S. (1986). The role of eyewitness confidence in juror perceptions of credibility. *J. Appl. Soc. Psychol.* 16 387–409. 10.1111/j.1559-1816.1986.tb01148.x

